# Augmentation of Electrocardiographic QRS R-Amplitude Precedes Radiocontrast-Induced Hypotension during Mobile Computed Tomography Scanning

**DOI:** 10.3390/jcm8040505

**Published:** 2019-04-12

**Authors:** Hye-Mee Kwon, Sung-Hoon Kim, Hee-Sun Park, Yong-Seok Park, Young-Jin Moon, Jae-Man Kim, Robert Thiele

**Affiliations:** 1 Biosignal Analysis and Perioperative Outcome Research Laboratory, Department of Anesthesiology and Pain Medicine, Asan Medical Center, University of Ulsan College of Medicine, 88, Olympic-ro 43-gil, Songpa-gu, Seoul 05505, Korea; hyemee.kwon@amc.seoul.kr (H.-M.K.); heespark@amc.seoul.kr (H.-S.P.); parkys@amc.seoul.kr (Y.-S.P.); yjmoon@amc.seoul.kr (Y.-J.M.); jaemankims@gmail.com (J.-M.K.); 2 Health Innovation Bigdata Center, Asan Institute for Lifesciences, 88, Olympic-ro 43-gil, Songpa-gu, Seoul 05505, Korea; 3 Departments of Anesthesiology and Biomedical Engineering, University of Virginia School of Medicine, Pinn Hall 1232, Charlottesville, VA 22908, USA; RHT7W@hscmail.mcc.virginia.edu

**Keywords:** contrast agent, computed tomography, electrocardiogram, QRS-R amplitude

## Abstract

Although intravenous administration of contrast media may trigger a variety of adverse reactions, sedated patients undergoing computed tomography (CT) scanning usually are not able to report their symptoms, which may delay detection of adverse reactions. Furthermore, changes in vital signs cannot be typically measured during mobile CT scanning, which worsens the situation. We aimed to characterize contrast-related hemodynamic changes that occur during mobile CT scanning and predict sudden hypotension based on subtle but robust changes in the electrocardiogram (ECG). We analyzed the digitized hemodynamic data of 20 consecutive patients who underwent clipping of a cerebral artery aneurysm and contrast-enhanced CT scanning following the surgical procedure. Hemodynamic variables, including ECG findings, invasive blood pressure (BP), pulse oximetry results, capnography findings, cardiac output, and systemic vascular resistance, were monitored simultaneously. We measured morphological changes in ECG-derived parameters, including the R–R interval, ST height, and QRS R-amplitude, on a beat-to-beat basis, and evaluated the correlation between those parameters and hemodynamic changes. After the radiocontrast injection, systolic BP decreased by a median 53 mmHg from baseline and spontaneously recovered after 63 ± 19 s. An increase in QRS R-amplitude (median 0.43 mV) occurred 25 ± 10 s before hypotension developed. The receiver operating characteristic curve showed that a 16% increase in QRS R-amplitude can predict a decrease in systolic BP of >25% (area under the curve 0.852). Increased cardiac output (median delta 2.7 L/min from baseline) and decreased systemic vascular resistance (median delta 857 dyn·s/cm^5^ from baseline) were also observed during hypotension. During mobile CT scanning, profound but transient hypotension can be observed, associated with decreased vascular resistance. Augmentation of QRS R-amplitude from an ECG represents a sensitive surrogate for onset of a hypotensive episode after contrast injection, thereby serving as a simple and continuous noninvasive hemodynamic monitoring tool.

## 1. Introduction

The intravenous administration of contrast media may trigger various kinds of adverse reactions such as nausea, dizziness, urticaria, and profound hypotension within minutes after administration [1,2]. It is speculated that an anaphylactoid reaction to peripheral injection of contrast agents plays a role in causing such a reaction, but the precise mechanism has not been clarified. In an effort to prevent such adverse events, prophylactic administration of pharmacological agents has been explored [1,3,4]. However, conclusive evidence is still lacking with doubtful results, and the titrating dosing does not seem to be effective since most anaphylactoid reactions do not seem to be dose-dependent.

The safety of contrast agents is widely reported, especially after introduction of non-ionic and low-osmolar iodinated agents, and adverse reaction incident rates are reported to be as low as 0.4–0.7% [1,5,6]. However, given the circumstances that the hemodynamic effect of contrast injection is transitory and usually followed by immediate recovery within 3-4 minutes [7,8], it is possible that unrecognized hypotension may have occurred under noninvasive blood pressure (BP) monitoring. Moreover, continuous monitoring is not generally performed as most patients have a short exposure duration to the contrast agent, which increases the possibilities of under-detection. Currently, detailed information of concurrent hemodynamic changes during computed tomography (CT) scanning is almost sparse. Furthermore, if patients are incapable of reporting their own symptoms, such as sedated patients undergoing CT scanning, it would more difficult to recognize the onset of an adverse event. Although most symptoms may be mild and transitory and recover without any complications, potentially life-threatening adverse events still occur. The important part of monitoring is that the attending physician should be able to detect any symptoms or signs in a timely manner, which could assist in early recognition and treatment [9].

This study aimed to document the characteristic hemodynamic changes during contrast-enhanced mobile CT scanning in anesthetized patients with simultaneous invasive BP and esophageal Doppler monitoring. Furthermore, we present the use of subtle changes in the electrocardiogram (ECG) as a noninvasive but robust index to predict hemodynamic changes.

## 2. Methods

This study was approved by the Institutional Review Board (IRB) of Asan Medical Center, and the requirement for written informed consent was waived by the IRB. We analyzed digitized hemodynamic data of 20 consecutive patients who underwent elective surgery of cerebral artery aneurysm clipping and had contrast-enhanced mobile CT scanning under general anesthesia following the surgical procedure. As part of our hospital’s protocol, hemodynamic variables, including ECG findings, invasive BP, pulse oximetry findings, and capnography findings, were routinely monitored during surgery. Eight patients were simultaneously monitored with an esophageal Doppler monitor (Cardio Q^®^, Deltex Medical Co., Chichester, UK), and cardiac output and systemic vascular resistance were obtained in those patients.

All patients were anesthetized according to the standard protocol of our institution [10]. Briefly, induction and maintenance of general anesthesia were achieved with target-controlled infusions of propofol and remifentanil using an infusion pump (TCI pump, Orchestra Base Primea, Fresenius Vial, France). The infusion rate was titrated to maintain adequate anesthetic depth, targeting a bispectral index between 40 and 60, as assessed with a BIS A-1050 Monitor (Aspect Medical Systems, Newton, MA, USA). Facilitation of muscle relaxation was achieved with rocuronium bromide (0.7 mg/kg) for tracheal intubation. Fresh gas flow was maintained at 3 L/min of medical air containing 50% oxygen. Mechanical ventilation was applied using a tidal volume of 6 mL/kg, with a respiratory rate of 8–10 breaths/min to maintain normocapnia during surgery. Phenylephrine was infused at a rate of 100-2500 mcg/h, titrated to maintain intraoperative BP at similar level to preoperative BP, if necessary.

After surgery, all patients underwent mobile CT scanning under general anesthesia in the operating room, using Pamiray370 (iopamidol 370 mgI/mL; Dongkook Pharma, Seoul, Korea), which is a non-ionic, low-osmolar iodinated contrast agent. The contrast agent was administered via the peripheral vein in all patients. For perfusion CT of cerebral angiography, a 45-mL bolus of contrast agent followed by a 90-mL bolus was injected, both at an infusion rate of 4 mL/sec. Beat-to-beat hemodynamic data including ECG findings, invasive BP, pulse oximetry results, and capnography findings were simultaneously recorded throughout the procedure by multi-wave data acquisition software (WINDAQ, DI-720U; DATAQ Instruments, Inc., Akron, OH, USA) at a 500-Hz sampling rate. In some patient (*n* = 8), the data from esophageal Doppler monitor were also simultaneously recorded because not all patients were indicated for Doppler monitoring. The ECG waveform and arterial waveform were retrieved from the electronically archived multi-wave data and transferred to Labchart^®^ 8 Pro (version 8.1.5, ADInstruments, Dunedin, New Zealand). In measuring the QRS R-amplitudes, the isoelectric line was defined as the PR interval. The beat-to-beat QRS R-amplitude and ST height were automatically measured from retrieved ECGs saved in the electronically archived multi-wave data. The Labchart^®^ software automatically determined the baseline of the ECG waveform, peak of the QRS R-amplitude, and start of the T wave. If automatically measured values were not acceptable by visual inspection, we manually adjusted the measurement. From the arterial waveform, we retrieved systolic, mean, and diastolic BPs, dP/dt max (mmHg/sec), and the contractility index (1/sec).

Hemodynamic data from each patient were compared at 4 different time points: (1) baseline, an average of 1 minute of data collected just before the increase of the QRS R-amplitude; (2) peak, the highest QRS R-amplitude after contrast injection; (3) nadir, lowest BP observed after contrast injection; and (4) recovery, BP recovers and returns to plateau status.

### Statistical Analysis

The data are presented as n (%), means ± standard deviation, or median (interquartile range), as appropriate. After testing for normality with the Shapiro-Wilk test, a paired *t*-test or Wilcoxon signed-rank test was used to compare parameters between the 4 time points, as appropriate. The receiver operating characteristic (ROC) curves and respective areas under the curve (AUC) were applied to evaluate ability of the QRS R-amplitude to predict a 25% decrease in the systolic BP (SBP). Both the QRS R-amplitude and SBP were obtained 5 consecutive times at peak and nadir points per patient (so-called cluster data). The AUC was computed with block bootstrap resampling that accounted for clustering of same patient. The block bootstrap replicates the correlation by resampling instead of blocks of data, as described elsewhere [11]. R version 3.4.2 software (R Foundation for Statistical Computing, Vienna, Austria) was used for all statistical analyses.

## 3. Results

The demographic and clinical characteristics of the enrolled patients are shown in Table 1. Patients’ mean age was 59.2 ± 8 years (5 men and 15 women). The mean left ventricular ejection fraction was 64.3 ± 2.6% (range 60–68%), and the mean arterial elastance was 2.33 ± 0.67 (range 1.45–4.13).

**Figure 1:**
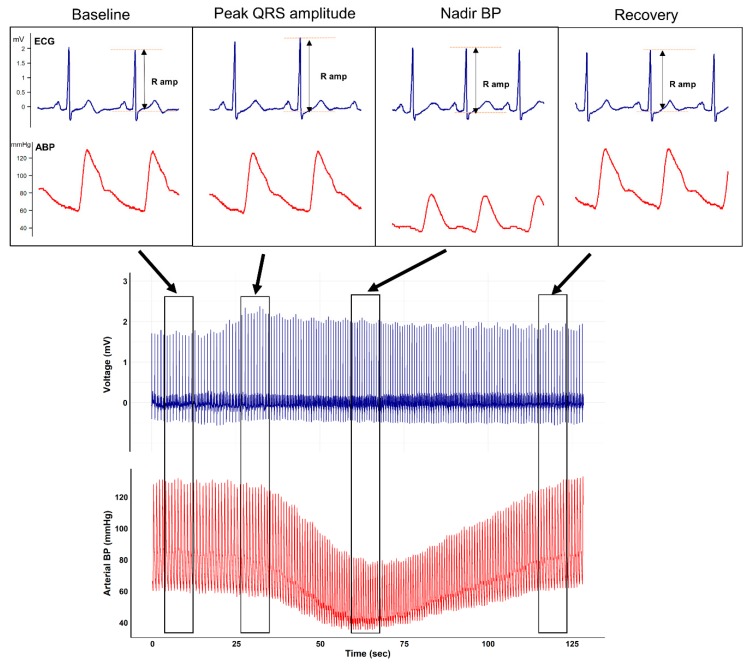
Representative beat-to-beat plot of the electrocardiogram and arterial waveform from an arbitrarily selected patient undergoing mobile contrast-enhanced computed tomography. After the injection of contrast agent, a characteristic QRS R-amplitude increase preceding nadir blood pressure is seen.

Representative plots of the ECG and arterial waveform during mobile CT scanning from an arbitrarily selected patient are shown in Figure 1.

**Figure 2:**
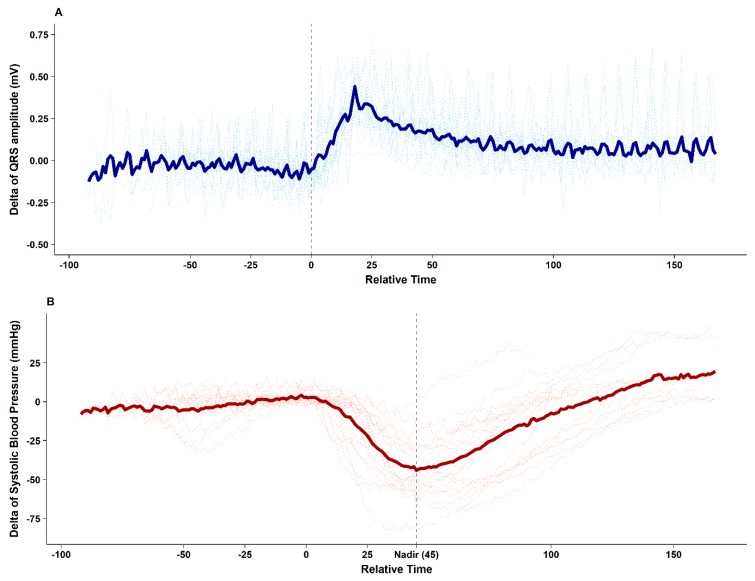
Delta changes from baseline of the QRS R-amplitude (**A**) and systolic blood pressure (**B**) after the injection of contrast agent during mobile computed tomography in all enrolled patients. An increase in the QRS R-amplitude (median 0.43 mV) is observed 25 ± 10 s before nadir systolic blood pressure. The median decrease in systolic blood pressure is 53 mmHg, which recovers to baseline level after 63 ± 19 s. The thick line indicates the mean value.

The figure clearly depicts that after contrast media injection, augmentation of the QRS R-amplitude preceded a profound decrease in BP. Collected QRS R-amplitude and SBP changes of all enrolled patients are depicted in Figure 2.

**Figure 3:**
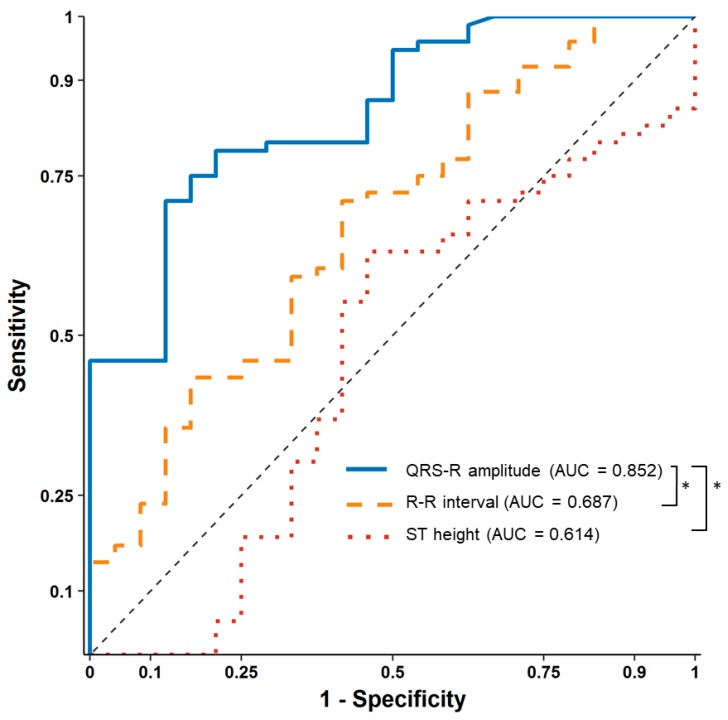
Receiver operating characteristic curve analysis of percentage changes in the QRS R-amplitude, ST height, and R–R interval in relation to ≥25% changes in systolic blood pressure. * Delong test, *p* < 0.001 compared to the QRS R-amplitude.

After contrast media injection, significant QRS R-amplitude augmentation (median of delta changes 0.43 mV, median 22% increase from baseline) was observed 25.2 ± 10.3 s before profound hypotension. The median decrease in SBP from baseline was 53 mmHg (median 40% decrease from baseline), which recovered after 62.7 ± 19.2 s. ROC curve analysis showed that 16% QRS R-amplitude augmentation from the baseline ECG can predict SBP drop > 25% with an AUC of 0.852 (95% confidence interval 0.774–0.929, *p* < 0.001), sensitivity of 71.1%, and specificity of 87.5%, whereas changes in the ST height (AUC 0.614) and R–R interval (AUC 0.687) were not significant during hypotension (Figure 3).

**Figure 4:**
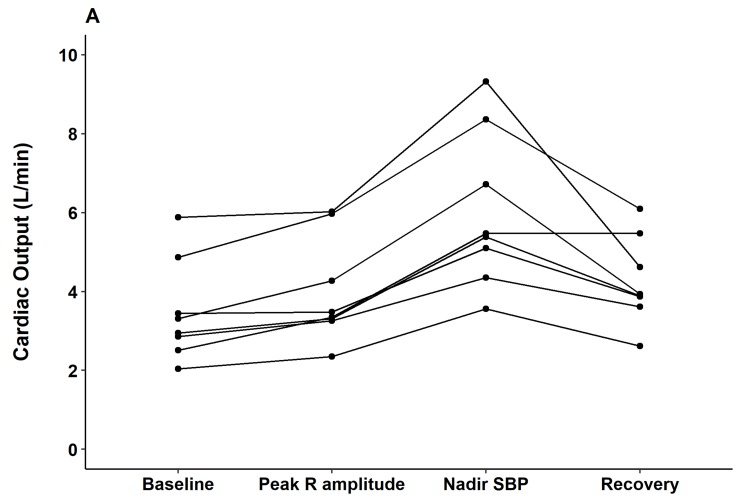

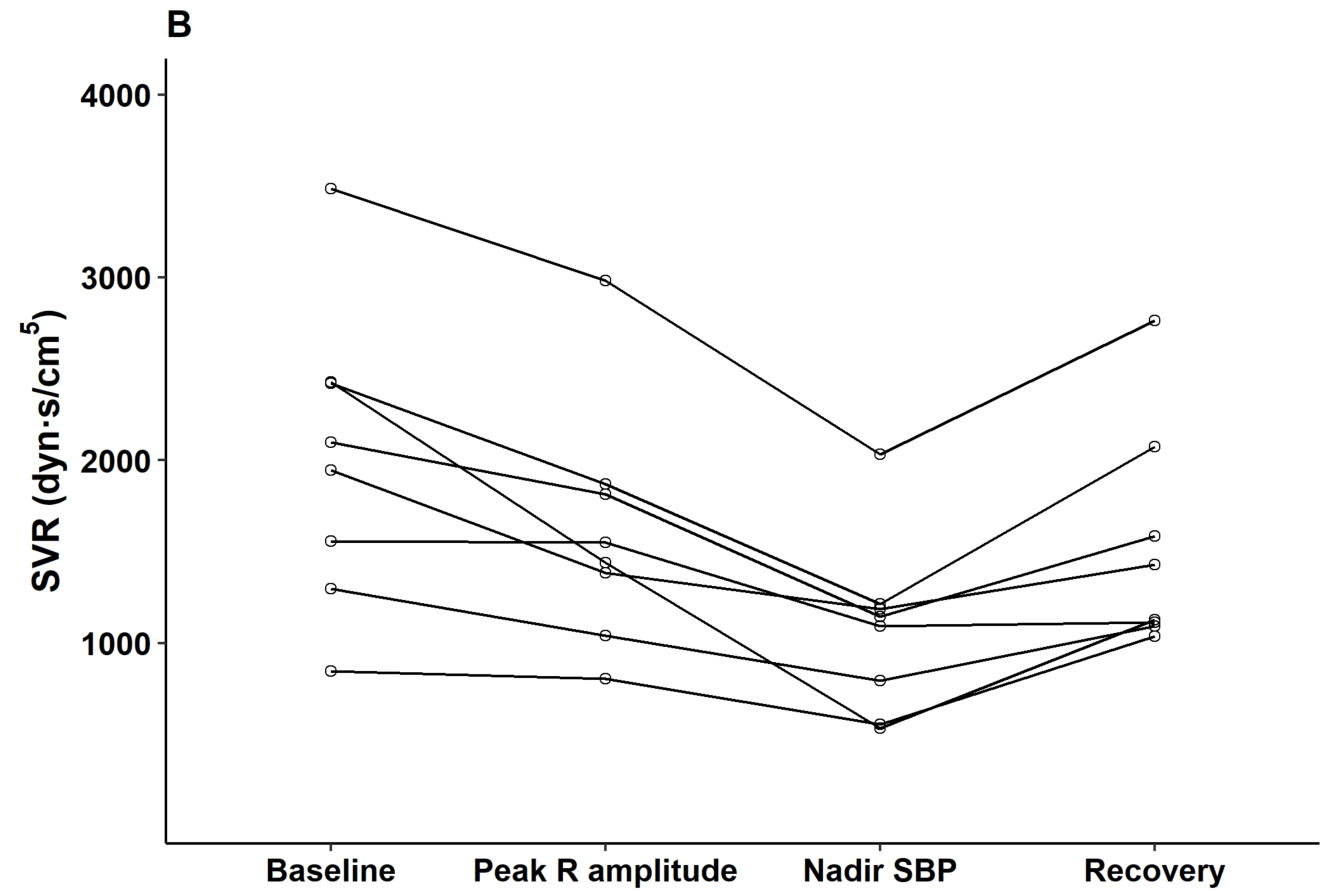
Changes in (**A**) cardiac output (L/min) and (**B**) systemic vascular resistance (dyn·s/cm^5^) from baseline to the peak QRS R-amplitude, nadir blood pressure (after contrast injection), and recovery for each subject (*n* = 8). * *p* < 0.05 compared to baseline.

The hemodynamic alterations were further investigated with arterial waveform analysis and esophageal Doppler monitoring. Contractile indices from the arterial waveform such as dP/dt max and the contractility index (1/sec) did not change from the time of contrast injection through the peak QRS R-amplitude period. However, significant decreases in those indices were observed at the nadir BP point (both *p* < 0.05). Parameters observed from esophageal Doppler monitoring revealed that after contrast injection and before nadir SBP, cardiac output gradually increased (median delta 2.7 L/min from baseline) while systemic vascular resistance gradually decreased (median delta 857 dyn·s/cm^5^ from baseline). From nadir SBP to the recovery period, cardiac output decreased while systemic vascular resistance increased to baseline level (Figure 4).

## 4. Discussion

The main result of the current study is that subtle but robust ECG changes preceded sudden hypotension after contrast media injection in patients undergoing contrast-enhanced CT scanning after cerebral artery aneurysm clipping. Specifically, augmentation of the QRS R-amplitude by 16% was observed 25.2 s earlier than the development of profound hypotension (25% decrease in SBP). This finding implies that morphologic changes in the ECG can be useful in detecting the onset of profound hypotension when invasive BP monitoring is not feasible. The hemodynamic assessment with esophageal Doppler monitoring showed that significant systemic vascular resistance decreased during hypotension.

Hemodynamic alteration is a well-known complication of intravenous contrast agent injection. An anaphylactoid reaction to peripheral injection of contrast agents is thought to be the cause of reported adverse events, but to date, the exact underlying mechanism remains uncertain [12]. It is speculated that the contrast agent activates mast cells and basophils, triggering the immediate release of histamine and nitric oxide [3]. Such changes may decrease systemic vascular resistance and be responsible for dramatic hypotension. After a certain point, the high concentration of generated nitric oxide has the potential to down-regulate histamine release and induce catecholamine generation, which may enhance myocardial contractility, contributing to the reversal of the decreased BP and even cause overshooting [7]. Our results are consistent with previous findings [3,7] that showed a gradual decrease of systemic vascular resistance during the decrease of BP (median 857 dyn·s/cm^5^ at nadir BP), and increase of dP/dt max and the contractility index at not before but during nadir BP and the recovery period (Table 2).

In the recent era, with the introduction of non-ionic and low-osmolar iodinated agents, a markedly reduced number of adverse events, as few as 0.4–0.7%, are reported, indicating a favorable safety profile [1]. However, previous reports focusing on the hemodynamic effect of contrast injection consistently showed transitory yet significant hypotension, followed by immediate recovery within 3-4 min [7,8]. In our study, all the enrolled patients experienced profound hypotension, although varying in degree, which recovered quickly (median 62.7 ± 19.2 s after nadir pressure). On the basis of previous [7,8] and current findings, we can suggest that hemodynamic changes may take place more than currently expected, and they may have been undetected because of their short duration. We are also able to speculate that well-known symptoms related to contrast agents, such as nausea/dizziness, may be associated with transient yet marked hypotension. Furthermore, in patients who have risk factors for fatal complications, such as those with heart failure, uncontrolled hypertension, or arrhythmia [1], timely detection of such hemodynamic instability may assist in prompt treatment. Given the short onset and duration of such hemodynamic changes, intermittent noninvasive BP monitoring may be too slow to detect such instability in a timely manner. However, considering the short period of exposure, continuous BP monitoring appears to not be appropriate because it is too invasive to be performed routinely. In the current study, we presented the characteristic QRS R-amplitude increase as a sensitive surrogate for the onset of profound of hypotension. Specifically, augmentation of the QRS R-amplitude by 16% from baseline could predict a >25% decrease in SBP with high sensitivity and specificity. Given that observation of preceding QRS R-amplitude augmentation before hypotension is feasible without additional monitoring devices, it highlights the efficiency of QRS R-amplitude augmentation not only as non-invasive but also as predictive surrogate.

Although the mechanism underlying electrocardiographic QRS R-amplitude changes has been thoroughly investigated, it continues to be elusive [13,14]. A mathematical model of electrical activity of the heart with theoretical analysis of electrocardiographic changes, including the QRS R-amplitude, was first postulated by Brody [13]. He suggested that the QRS R-amplitude has a dose-dependent relationship with the corresponding intracardiac volume. Thereafter, the QRS R-amplitude was suggested to detect coronary artery disease and heart failure in patients, on the basis of an increase in the ischemia-induced intracardiac volume [15,16]. However, an inconsistent relationship reported in other studies raised questions as to whether QRS R-amplitude serves as an appropriate surrogate of intracardiac volume [17]. Interestingly, a phenomenon showing QRS-R wave amplitude augmentation during hemodialysis had been consistently reported [14,18]. Although intense investigation has spanning almost 30 years to reveal determinants of such changes, the underlying mechanism has not been clarified; it has only been speculated that the change may be influenced by multifactorial factors [14].

In the current study, we found a characteristic QRS R-amplitude augmentation preceding profound hypotension after contrast injection. To our knowledge, this phenomenon has not been reported in previous studies, and the underlying mechanism has not been discussed. Currently, we can only speculate possible mechanisms. Firstly, in line with the Brody’s theory [13], an augmented QRS R-amplitude may be the reflection of sudden increase of stroke volume. In our results, an increase in stroke volume was observed through esophageal Doppler monitoring after contrast injection. However, the stroke volume continuously increased, whereas the QRS R-amplitude showed only a transient increase. Secondly, previous studies reported transiently decreased blood calcium levels due to a calcium chelating agent in radio-opaque compounds [19]. We can speculate that this alteration may affect depolarization and repolarization, which could result in an increase in QRS R-amplitude.

Although the underlying mechanism is a topic of future research, our result still highlights the usefulness of the QRS R-amplitude, a continuous noninvasive parameter, for predicting marked hemodynamic changes caused by a contrast agent. The efficacy of a noninvasive index when invasive hemodynamic monitoring is not feasible has been demonstrated before, such as use of the pulse transit time, calculated with photoplethysmography and electrocardiography, to track BP perturbation [20]. In living-donor transplantation, ventricular arrhythmogenic potential (prolongation of corrected QT, Tp-e interval, and Tp-e/QTc ratio) developed immediately after portal vein unclamping before the occurrence of systolic hypotension [21]. The usefulness of the QRS R-amplitude lies in that it not only tracks hypotension but can also be used to predict such alterations. Based on our results, the QRS R-amplitude increased 25.2 ± 10.3 s earlier than development of hypotension. This could allow attending physicians to recognize and be prepared for forthcoming hemodynamic instability, especially in hemodynamically vulnerable patients.

There are several limitations to the present study. First, we observed this particular phenomenon in anesthetized patients who underwent neurosurgery. As some special susceptibility may exist in the current result, further studies are warranted in a larger population of patients with different etiologies. Therefore, careful consideration should be taken when generalizing our results to other population Second, we are unable to elucidate the definite underlying mechanism causing the current phenomenon, as mentioned earlier. To our knowledge, not only QRS R-amplitude increase but also transient yet dramatic hypotension following contrast agent injection have been scarcely reported. Further prospective study on animal models will be needed to investigate such a phenomenon and its underlying mechanism.

In summary, the QRS R-amplitude from beat-to-beat ECG data represents a sensitive surrogate for onset of profound hypotension after contrast injection, thereby providing a simple and continuous noninvasive hemodynamic monitoring tool.

## Figures and Tables

**Table 1:** Demographics of patients undergoing contrast-enhanced computed tomography (*n* = 20).

Characteristics	Values
Age (years)	59.2 ± 8
Sex (male/female)	5/15
Height (cm)	159.8 ± 8.6
Weight (kg)	62.8 ± 14.6
Body mass index (kg/m^2^)	24.3 ± 3.5
Hypertension, n (%)	8 (40)
Diabetes mellitus, n (%)	1 (5)
Calcium channel blocker, n (%)	7 (35)
Angiotensin II receptor blocker, n (%)	6 (30)
Type of cerebral aneurysm	
MCA aneurysm	8 (40%)
A-com aneurysm	3 (15%)
P-com aneurysm	2 (10%)
Paraclinoid aneurysm	2 (10%)
Multiple aneurysms	5 (25%)
Preoperative echocardiographic finding (*n* = 17)	
Left ventricular ejection fraction (%)	64.3 ± 2.6
Left ventricle internal dimension at end diastole (mm)	47.5 ± 5.2
Left ventricular posterior wall thickness at end diastole (mm)	9.4 ± 1.2
Interventricular septal dimension at end diastole (mm)	8.6 ± 1.1
E/E’ ratio ^a^	9.7 ± 2.0
Arterial elastance	2.33 ± 0.67
Ventricular elastance	4.16 ± 1.04
Ventriculo-arterial coupling	0.56 ± 0.06

Data are presented as a mean ± standard deviation or number (percentage) ^a^ Ratio of early transmitral flow velocity to early diastolic velocity of the mitral annulus. MCA, middle cerebral artery; A-com, anterior communicating cerebral artery; P-com, posterior communicating cerebral artery.

**Table 2:** Electrocardiogram and hemodynamic variables before, during, and after contrast-enhanced computed tomography.

	Baseline ^a^	Peak QRS Amplitude	Nadir BP	After Recovery
Heart rate (beats/min)	52.8 ± 10.3	55.4 ± 9.7 *	63.7 ± 10.8 **	57.7 ± 10.9 **
QRS-R amplitude (V)	2.79 ± 1.04	3.29 ± 1.03 **	2.99 ± 1.06 **	2.91 ± 1.04 **
QRS interval (msec)	70.1 ± 12.7	74 ± 13.2 **	71.5 ± 12.9 ^†^	73.5 ± 15.1 *
ST height (mV)	−89.8 ± 134.6	−103.1 ± 127.7 ^†^	−70.6 ± 145.7 ^†^	−106.1 ± 139.3 *
Systolic blood pressure (mmHg)	125.6 ± 13.8	114.4 ± 26.1 ^†^	75.8 ± 19.8 **	125.9 ± 13.4 ^†^
Mean blood pressure (mmHg)	86.0 ± 8.8	79.5 ± 14.8 *	54.1 ± 12.6 **	87.1 ± 9.7 ^†^
Diastolic blood pressure (mmHg)	61.6 ± 7.0	55.2 ± 11.5 *	37.6 ± 9.1 **	62.2 ± 7.5 ^†^
dP/dt max (mmHg/sec)	1006.8 ± 152.6	969.4 ± 302.0 ^†^	819.5 ± 387.8 *	949.4 ± 174.4 *
Contractility index (1/sec)	12.02 (10.46–13.10)	12.27 (10.61–14.99) ^†^	14.81 (12.67–17.61) **	10.38 (8.89–11.73) *
Time from the peak QRS amplitude (sec)	−15.9 ± 6.1	0	25 ± 4.0	59.2 ± 17.4
Cardiac output (L/min) ^§^	3.5 ± 1.3	4.0 ± 1.3 *	6.0 ± 2.0 **	4.3 ± 1.1 ^†^
Stroke volume (mL) ^§^	66.5 ± 16.8	73.0 ± 15.4 *	93.3 ± 17.8 **	75.9 ± 14.4 ^†^
Systemic vascular resistance (dyn·s/cm^5^) ^§^	2010.2 ± 810.6	1611.0 ± 659.7 *	1146.5 ± 459.8 *	1528.4 ± 609.5 *

* *p* < 0.05 from baseline; ** *p* < 0.001 from baseline; ^†^ *p* > 0.05 from baseline; ^a^ Average of 60 s collected before an increase of the QRS amplitude. Data are presented as a mean ± standard deviation or median [interquartile range] as appropriate. ^§^ Values were derived from the CardioQ (*n* = 8).
